# Accelerometer-Based Method for Extracting Respiratory and Cardiac Gating Information for Dual Gating during Nuclear Medicine Imaging

**DOI:** 10.1155/2014/690124

**Published:** 2014-07-10

**Authors:** Mojtaba Jafari Tadi, Tero Koivisto, Mikko Pänkäälä, Ari Paasio

**Affiliations:** ^1^Technology Research Center, Brahea Center, University of Turku, 20520 Turku, Finland; ^2^Department of Clinical Physiology and Nuclear Medicine, Faculty of Medicine, University of Turku, 20520 Turku, Finland

## Abstract

Both respiratory and cardiac motions reduce the quality and consistency of medical imaging specifically in nuclear medicine imaging. Motion artifacts can be eliminated by gating the image acquisition based on the respiratory phase and cardiac contractions throughout the medical imaging procedure. Electrocardiography (ECG), 3-axis accelerometer, and respiration belt data were processed and analyzed from ten healthy volunteers. Seismocardiography (SCG) is a noninvasive accelerometer-based method that measures accelerations caused by respiration and myocardial movements. This study was conducted to investigate the feasibility of the accelerometer-based method in dual gating technique. The SCG provides accelerometer-derived respiratory (ADR) data and accurate information about quiescent phases within the cardiac cycle. The correct information about the status of ventricles and atria helps us to create an improved estimate for quiescent phases within a cardiac cycle. The correlation of ADR signals with the reference respiration belt was investigated using Pearson correlation. High linear correlation was observed between accelerometer-based measurement and reference measurement methods (ECG and Respiration belt). Above all, due to the simplicity of the proposed method, the technique has high potential to be applied in dual gating in clinical cardiac positron emission tomography (PET) to obtain motion-free images in the future.

## 1. Introduction

Motion artifacts decrease the quality of the nuclear medicine imaging. Motion artifacts may lead to incorrect diagnosis, unnecessary treatment, and insufficient therapy [1]. The sources of motion cause artifact through the positron emission tomography (PET) or computed tomography (CT) procedures on acquired images. The motion might cause blurred and imperfect images in myocardial perfusion images as well as incorrect assessment of tumor size and activity in oncological imaging such as lung metastasis imaging [2]. Therefore, motion artifacts are the major concern in medical imaging especially in nuclear medicine imaging (PET/CT) [3]. In order to get high quality outcomes, cardiac images need to be obtained when the heart is in a quiescent phase inside a cardiac cycle. ECG is an outstanding instantaneous forecaster of electrical state and average mechanical state, but, due to the stirring movements of the heart, ECG fails to trace the instantaneous mechanical state of heart [4]. Broadly studied techniques to improve temporal resolution and reduce the motion artifacts include cardiac gating utilizing the ECG gating, respiratory gating, and dual gating. These techniques are mainly based on the investigation of electrical state of the heart, the chest wall movement, and the analysis of emission data in clinical studies, respectively [1, 4–6].

That is, by means of synchronizing the image acquisition or reconstruction with physiological motions, the motion artifacts can be reduced [7]. However, due to some drawbacks such as imperfect temporal resolution in cardiac imaging, supplementary stress on patients, and unsatisfactory patient comfort during long data processing time, none of the mentioned gating procedures has acquired stable status in the clinical procedure of nuclear medicine imaging [1].

Seismocardiography (SCG), which is an accelerometer-based method, can be obtained by means of an accelerometer sensor located on the sternum of the subject [4, 8–13]. SCG measures accelerations caused by respiration and myocardial movements in the chest wall using a microelectromechanical sensor (MEMS) mounted on the chest of the subjects [14]. The SCG provides accelerometer derived respiratory data and the accurate information about quiescent phases within cardiac cycle according to the chest wall movement and mechanical states of the heart, respectively. The accelerometer-based method can be used to assess the mechanical activity of the heart by detecting aortic opening and aortic closure events from the SCG signal [15, 16]. The correct information about the status of ventricles and atria helps us to create an improved estimation of quiescent phases throughout a cardiac cycle [4, 8–10]. This study focuses on the extraction of the cardiac and respiration signals from SCG signal which could be valuable for nuclear medicine imaging. The purpose of the study is to develop a method of extracting precise peak-to-peak timing location of cardiac systole and diastole. These locations are extracted using mechanical aortic opening (AO) and aortic closure (AC) points visible in the seismocardiogram signal. Additionally, the correlation between respiratory cycle durations of accelerometer derived respiration (ADR) and reference respiration belt will be evaluated. In this case, assessment of the potential of the SCG signal as a gating signal for dual gating will be evaluated. Naturally, suitable filtering is required in order to remove out-of-band components. This study is accomplished using the Matlab programming environment (MathWorks, Inc.).

## 2. Materials and Methods

### 2.1. Human Subject Protocol

This study was implemented by the Technology Research Center (TRC) Laboratory at the University of Turku, Finland. The test group of this study consisted of ten healthy volunteers from different ages, heights, weights, and body mass indexes (BMI).

The respiratory measurements were performed using a predefined three-phased breathing pattern. This protocol was defined to investigate the accuracy of the accelerometer derived respiratory signal during three different breathing types as well as detecting the breathing rate versus the reference respiration belt signal. The breathing protocol consisted of 2 min normal breathing, 1 min slow breathing, and 1 min fast breathing continuously. During each section, subjects were asked to utilize mostly their lungs for breathing. The respiratory characteristics of the ADR signal were conducted to compare with the respiration belt signal. In addition, ECG signal was used as a reference signal for electrical characteristics of the heart.

The demographics of the subjects were as follows (min–max, mean, and standard deviation): age (23–38, 27.6, and 4.81 years), height (173–190, 179.35, and 5.02 cm), weight (65–85, 74.8, and 7.69 kg), and BMI (20.7–25.7, 23.2, and 1.54 kg m^−2^).

**Figure 1:**
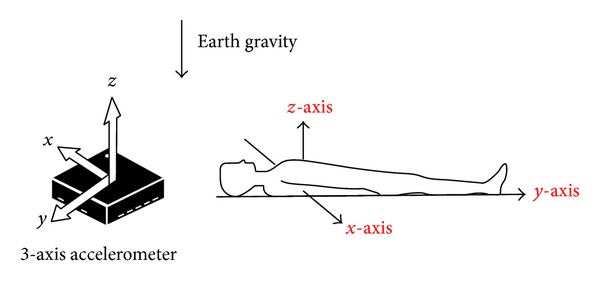
Direction of the detected accelerations from the subjects.

Figure 1 shows the acceleration axes, *y*, *x*, and *z*, which are longitudinal element (foot-head), lateral element (left-right), and sagittal element (dorsoventral), respectively. Besides, the test subjects were asked to lay supine and placed their hands next to their bodies on a camping bed with the accelerometer attached to their chest and respiration belt fastened around the upper chest.

Validity of the ADR signal against the respiration belt signal was evaluated using Pearson correlation function (similarity of waveform morphology). Pearson correlation coefficient was calculated to evaluate the linear relationship between the accelerometer-based measured results and reference measurements with all the test subjects. Besides, Bland and Altman plots were used to evaluate the agreement among the cardiac intervals extracted from the SCG and ECG as well as respiratory cycle durations across the ADR and respiration belt signals [17].

### 2.2. Measurement System

**Figure 2:**
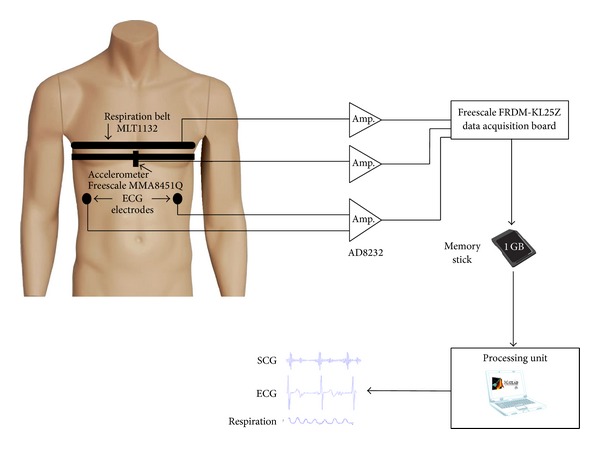
Block diagram of measurement setup. The ECG electrodes, accelerometer, and respiration belt were connected to the main measuring unit. After that, the collected data were transferred to the processing unit which was a personal computer.

Figure 2 shows a block diagram of the measurement system. SCG, ECG, and respiration belt data were recorded simultaneously using a custom-made acquisition system with a sampling frequency of 800 Hz. A (3 mm × 3 mm × 1 mm) triple-axis, low-power, capacitive digital accelerometer (Freescale Semiconductor, MMA8451Q, Austin, TX, USA) with 14 bits of resolution was attached to the body of sternum using a narrow elastic belt.

The design of the measurement system, data acquisition, and postprocessing procedures of this study were accomplished in the TRC laboratory at the University of Turku, Finland. The data acquisition involved measurement of lungs' movements, acceleration (three axes of measurement), and ECG using Freescale FRDM-KL25Z board to collect the data on a memory stick. The respirometry components were obtained using a piezoelectric respiratory belt transducer (ADInstruments, MLT1132, Dunedin, New Zealand) attached to an elastic belt that measures the variations in the thoracic circumference. The belt was fastened around the middle part of the rib cage of the subjects to obtain thoracic breathing. The accelerometer was mounted on the body of sternum using a narrow elastic belt located somewhat lower than the respiration belt. The ECG measurements were accomplished utilizing two electrodes on the anterior lateral regions to the abdomen at the left and the right hypochondriac region of the subjects' body (Figure 2). The electrodes were standard Blue Sensor M ECG electrodes (Ambu, Ballerup, Denmark). Electrode sites were made with wet gel to make excellent signal quality during the test. During this study, only one electrocardiogram was utilized as a reference ECG signal. The ECG signal was simultaneously recorded using a single lead, Heart Rate Monitor Front-End (Analog Devices, Inc., AD8232, MA, USA). The reference respiration, accelerometer, and ECG data processing were accomplished after transferring the binary data from the memory card to a computer to convert the data to Text Format (.txt) using a custom-made software. The obtained text file was well-matched with the Matlab programming environment (MathWorks, Inc.) for the offline analyzing of the acquired signals.

### 2.3. Signal Processing

**Figure 3:**
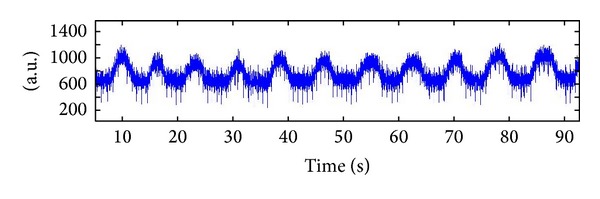
A raw SCG signal was acquired from the measurement system.

According to Figure 3, two components were dominant in the SCG signal as follows:

1. a large low-frequency (sub-Hz) element due to movement of the chest wall caused by expansion and contraction of the lungs during the test;
2. a component with sharper amplitudes and upper frequency (>5 Hz) contents caused by pulsations of the chest wall due mainly to acoustic waves produced by the heart valves as reported by [18].

Figure 3 demonstrates the raw signal extracted from SCG which contains these two main components (cardiac and respiratory signals).

#### 2.3.1. Cardiac Signal Processing

**Figure 4:**
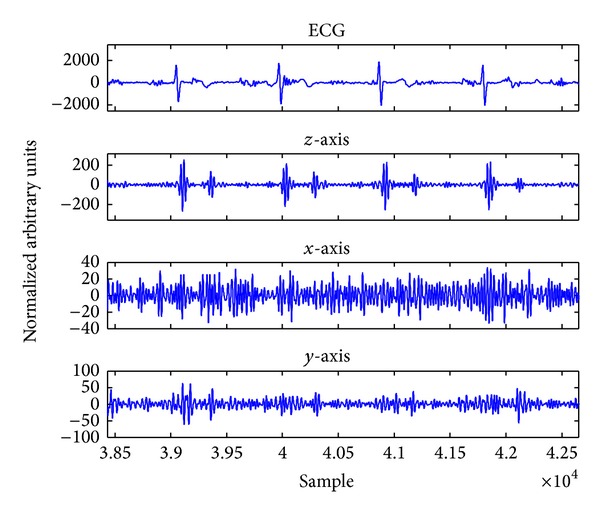
Demonstration of filtered ECG signal and accelerometer signals from different axes.

All of the acquired data were normalized and zero averaged according to the signals' standard deviation to be formulated for direct assessment. All the three acceleration signals extracted from each axis plus ECG were filtered using a fast Fourier transform (FFT) filtering method. The FFT band-pass filter was designed with the cut-off frequencies of 20 Hz and 40 Hz, respectively. The ECG signal was filtered with the cut-off frequencies of 1 Hz and 40 Hz, respectively. The FFT filter was used to remove high frequency noises caused by various sources of noise.  Figure 4 indicates ECG signal associated with three axes of SCG data extracted from the measuring system. Unlike ECG, the SCG is affected by the anatomical characteristics so that there are significant differences in the acquired data. According to Figure 4, the *z*-axis has less distortion in comparison with the ECG which has made it a sufficient signal to investigate the cardiac cycles. As can be seen from this figure, the locations of the *z*-axis peaks were much closer to the R peaks of QRS complex than *y*- and *x*-axes. Furthermore, *z*-axis components have higher amplitude in comparison with components of the two other axes. As demonstrated earlier by Figure 1, the *z*-axis is coaxial with the lungs movement and the heart. Thus, because of locating the accelerometer in this position (coaxial with the lungs and heart), the *z*-axis has acquired the cardiac microvibrations better than the other axes.

The first breathing part consisted of 2-minute normal breathing. This part was analyzed for peak-to-peak analysis of SCG and ECG signals with all test subjects. Likely, due to some distortions at the beginning of measurements, a few seconds of the SCG, ECG, and respiration belt measurements were omitted by the same size. However, detecting the precise location of AO and AC points across the SCG signal was one of the main challenges of this study. Different methods were examined to detect the right positions of AO and AC, but the windowing method provided reliable results to find these points.

**Figure 5:**
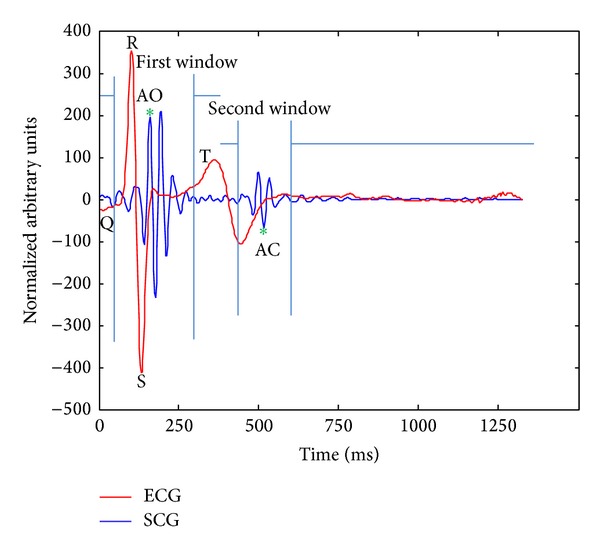
The windowing technique for detecting AO and AC peaks.

Figure 5 represents the basis of windowing method in time domain. Since detection of the R peaks across the ECG signal was straightforward, the R peaks were found applicable to be used as reliable time points for detecting the cardiac events throughout the SCG signal. Thus, detection of R peaks allowed us to detect the AO peaks by using time-domain windows before and after each R peak. The length of the windows varies case by case based on the heart rate of the subjects. Across the first taken window, the maximum value of the signal was calculated in order to find the AO peak. Then, the second window was taken few hundred milliseconds before and after the next R peak and the current detected AO, respectively. The length of the second window was longer than the first one due to uncertainty to find the exact location of the AC time point after AO point. The AC peaks were detected by calculating the minimum value of the signal within the taken window based on what [4] has determined for the location of AC. With detection of the exact locations of cardiac phases, reliable precise measurements were performed to obtain the electromechanical cardiac intervals such as R-R, R-AO, R-AC, AO-AO, and AO-AC intervals.

The AO and AC peaks were detected like a dip-rise-dip wave and a rise-dip-rise wave caused by cardiac contraction and relaxation, respectively. However, in some cases there was some variation in amplitude of AO and AC. In this case, developing a reliable algorithm which is able to identify various situations of acoustic sounds of the heart caused by opening and closure of the aortic valve was required. Furthermore, in order to represent an accelerometer-based solution for cardiac gating, the gating algorithm is required to detect cardiac intervals properly as well as assess the applicability of the intervals for the cardiac gating based on acceptable heart rate variability range.

#### 2.3.2. Respiratory Signal Processing

Approximately, 240 s of the respiration belt and accelerometer data including all three breathing patterns was analyzed with all test subjects. Due to various frequency components caused by three different breathing patterns through the respiratory signals, the FFT filter did not perform properly to extract the low-frequency components (accelerations caused by heart movements) of SCG. Therefore, both respiration belt and SCG signals were downsampled to the half (sampling frequency was 800 Hz). Then by implementing digital filters, the raw SCG signal was filtered. With both moving-average and one-dimensional median filters, the sharp and high frequency picks of SCG signal were removed out.

It should be mentioned that the number of points (order) for 1D median and moving-average filters was defined based on the nature of the outcome signal from the filter. Obviously, due to different morphological features of cardiac and respiratory signals extracted from each test subject, it made us try different orders for each filter. Therefore, an individual set was applied for median and moving-average filters to produce a neat and noiseless signal.

Unlike *z*-axis data, *x*- and *y*-axes data were not disturbed as much as *z*-axis by the heart beats. The acquired ADR signal from each axis demonstrated that *x*- and *y*-axes acquired the respiratory signals better than the *z*-axis.

In contrast, *z*-axis measured the accelerations caused by the acoustic sounds of the cardiac valves better than the other axes. Therefore, the importance of utilizing the triaxis accelerometer for the simultaneous extraction of the cardiovascular signals was recognized based upon the aforementioned points.

Respiratory phases (normal, slow-paced, and fast-paced) were determined using a peak detection algorithm which was sensitized to the minimum peak height and minimum separation distance between the peaks of both ADR signal and respiration belt. The ADR signal was also evaluated against the respiration belt signal in which only the respiratory cycle durations (peak-to-peak) from all breathing patterns were investigated by Pearson correlation. The correlation test between respiratory cycle durations was implemented to assess the linear relationship between the length of breathing cycles across the ADR and respiration belt signals. Respiratory cycle length is a significant factor (e.g., for determining Bin duration) for dual gating where the respiration signal should be divided into distinct phases to be synchronized with cardiac signals [21].

**Figure 6:**
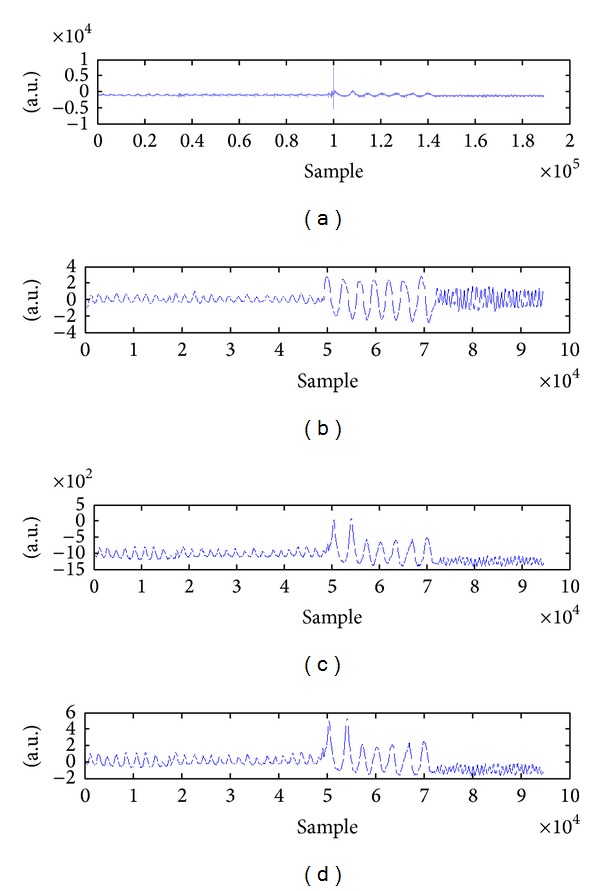
Process of extracting the ADR signal from the raw SCG data.

Figure 6 shows the aforementioned process for extracting respiratory signal from SCG signal. As shown in this figure, Figures 6(a) and 6(b) refer to the raw SCG signal with sampling frequency 800 Hz and reference respiration belt signal with sampling frequency 400 Hz, respectively. Figures 6(c) and 6(d) demonstrate the extracted respiratory signal after the 1D median filter and moving-average filter with sampling frequency 400 Hz, respectively.

## 3. Results

A high quality mechanical signal was obtained after filtering the raw SCG data from the *z*-axis with ten subjects (Figure 6(b)). The electrical signal of the heart was extracted using the reference ECG as well (Figure 6(a)). Mechanical signals of the *y*-axis and *x*-axis were filtered; however, they were not considered for cardiac measurements in this study (Figures 6(c) and 6(d), resp.). The cardiac phases of SCG were correlated well with the cardiac events across the ECG with a small delay.

The accelerometer-based measurements were assessed versus the ECG measurements. The assessment was based on the evaluation between the cardiac event intervals of ECG and SCG. The AO and AC points were detected using the advanced algorithm which was developed for accurate peak detecting across the SCG signal. From the ECG signal, the R peaks were detected by the same algorithm that was used to detect respiratory phases and breathing rate. Although it was possible to analyze both cardiac signals (ECG and SCG) totally, only the first 120 s of SCG signal (calm breathing) per test subject was investigated in order to acquire representative results.

Assessment of correlation and agreement between the measured information and reference data was performed under the standard circumstances. Pearson correlation was used to assess the linear relationship between cardiac intervals extracted from the SCG and ECG. The correlation between the length of R-R and AO-AO intervals was very high (0.9903–0.9998). Also high correlation (0.6–0.93) between the length of AO-AC and R-R intervals was seen across the SCG and ECG signals.

**Figure 7:**
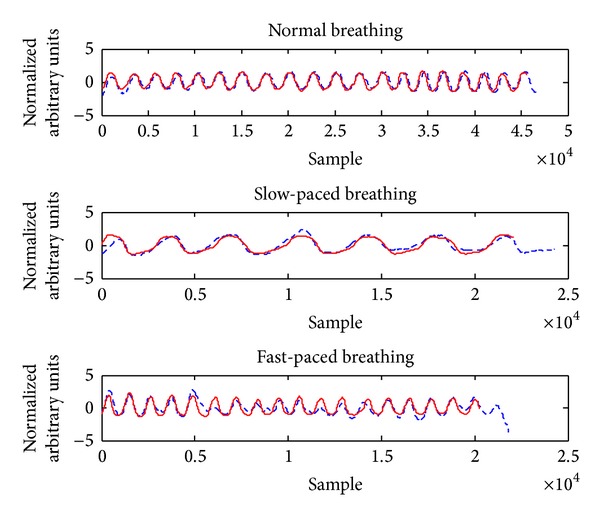
Demonstration of the ADR signal (dashed line) which is following well the reference signal in different breathing patterns.

A respiratory signal was extracted from the unprocessed SCG data. As Figure 7 demonstrates, the ADR signals are following well the reference respiration belt components during different breathing patterns by every person (Figure 7). Different breathing rates were observed for each subject in each breathing pattern.  Table 1 indicates the mean and standard deviation of the breathing rate during each pattern from ADR signal with all the test subjects. It can be seen from Table 1 that standard deviation in the fast breathing is quite high. That is, subjects did not understand the required speed of fast-paced breathing. This factor shows that every test subject had different breathing rate in fast-paced breathing and they have not breathed based upon any instruction to control their breathing depth.

The correlation coefficient (*r*) between ADR and respiration belt measurements was very high in all respiratory cycle durations. Pearson correlation coefficient values were obtained as 0.9945, 0.9981, and 0.9941 for normal breathing, slow-paced breathing, and fast-paced breathing, respectively. Figure 7 indicates the correlation of the breath signals extracted from accelerometer and reference respiration belt with three breathing patterns (normal, slow-paced, and fast-paced). As illustrated in Figure 7, ADR signal (red dashed line) has high correlation with the reference respiration belt (red solid line).

As shown in Table 1 the standard deviation of the subjects in normal and slow-paced breathing was 1.73 and 1.83, respectively. It means that the subjects with different heart rates had a maximum breath rate between 9 and 12 bpm which is important, because the breathing rate variation during the scan is a potential source of error. Therefore, with the regular breathing rate the amount of the error (motion artifacts) will be minimized. That is, the lower the breath rate, the lower the motion errors [19].

## 4. Discussions

The aim of this study was to develop an accelerometer-based dual gating method for nuclear medicine imaging by detecting and segregating of the cardiac and respiratory motion signals instantaneously. A hardware system which was able to obtain synchronous seismocardiography, electrocardiography, and respiration belt data was used to implement this study. The system was designed in the Technology Research Center, Brahea Center, University of Turku.

This research investigated whether a 3-axis accelerometer has the potential to prepare the dual gating method for the cardiac PET imaging. This study concentrated on validating the accelerometer-based measurement against different reference methods (respiration belt and ECG). As a result, this method represented that cardiovascular information obtained from accelerometer has high linear correlation with the reference measurements. In addition, agreement between the accelerometer and reference methods was found to be good in cardiac intervals and respiratory cycle durations; however, there is some variation in the differences between the methods. Nevertheless, the majority of the differences deviate less than 10% of the mean values of cardiac intervals. The current method measured all cardiac intervals only with subjects having a fairly normal sinus rhythm.

This study investigated the linear correlation between accelerometer-based respiratory and reference respiratory measurements. Furthermore, this study assessed the agreement between the accelerometer derived respiratory (ADR) and reference respiration signal in respiratory cycle duration (peak-to-peak interval measurements).

**Figure 8:**
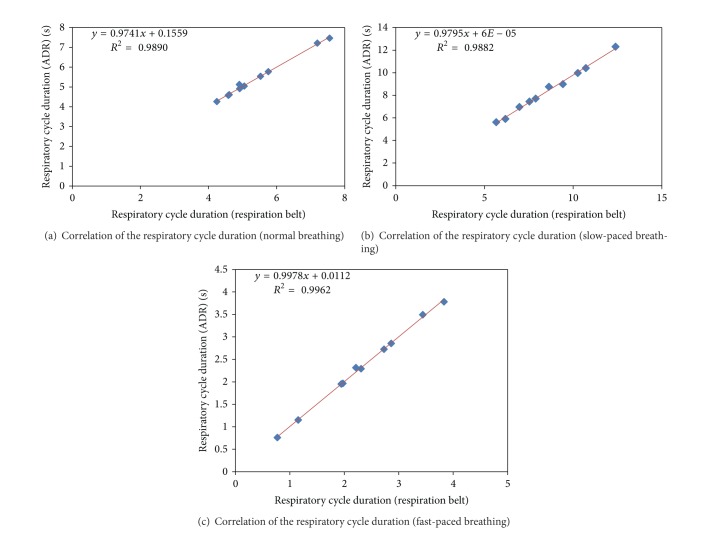
Assessment of correlation between the ADR and reference respiration signal shows a high correlation in regard to respiration cycle duration across normal breathing (a), slow-paced breathing (b), and fast-paced breathing (c).

As results were shown in this study, a high linear correlation was observed between accelerometer-based measurement and reference measurement approaches (ECG and respiration belt). According to Figure 8, the ADR respiratory cycles correlate well with the reference respiration belt respiratory cycles. Figures 8(a), 8(b), and 8(c) display that the time-based accuracy and correlation of ADR method were very high concerning the reference respiration belt in each breathing mode. Thus, this study verifies that accelerometer-based method has high potential to implement the respiratory gating using accelerometer-based derived respiration signal.

Furthermore, accelerometer-based measurement is a precise technique for detection of temporal cardiac events which implies the potential of the accelerometer-based method to be used in cardiac gating. However, there are some differences between the current equipment (ECG, SCG, real-time position management (RPM) system, and ADR system) for dual gating. Indeed, further study is needed to determine this technique as a standard method for patient study by real dual gating.

**Figure 9:**
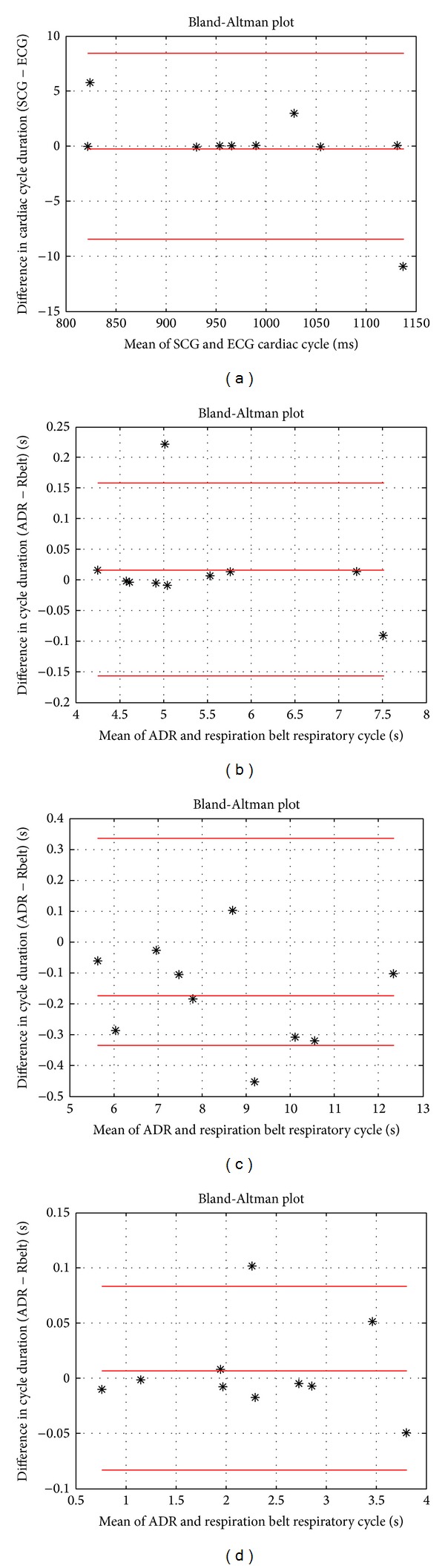
Bland-Altman plots of accelerometer-based measurements. (a) High agreement between SCG and ECG cardiac cycle duration. ((b), (c), and (d)) Good agreement of the respiratory cycle duration across normal, slow-paced, and fast-paced breathing, respectively.

Figure 9(a) indicates the agreement between the SCG and ECG across 10 subjects. The Bland Altman diagram for the cardiac cycle durations obtained from SCG signals, relative to ECG, is shown in Figure 9(a). The bias value is −0.24 (95% confidence levels: upper 8.2, lower −8.2) for SCG. On this basis, the agreement between the accelerometer-based method and electrocardiography was found very high.

The agreement among the methods in respiratory cycle duration was very high. Figures 9(b), 9(c), and 9(d) indicate the Bland-Altman plots in relation to the agreement analysis for respiratory cycle duration across normal, slow-paced, and fast-paced breathing, respectively. As shown in Figure 9(b), the confidence interval for respiratory cycle duration during normal breathing was obtained to lie within ± 0.1724 with a bias of 0.0152. Besides, as can be seen from Figure 9(c), the bias value is −0.1758 (95% confidence levels: upper 0.35, lower −0.35) for slow-paced breathing. Finally, the confidence interval for respiratory cycle duration, computed across the fast-paced breathing with all the subjects, was found to lie within 0.0894 and −0.0774 with the bias of the 0.006 (Figure 9(d)). Consequently, according to the aforementioned results, good agreement between the ADR and reference respiration belt as well as ECG and SCG methods was detected.

**Figure 10:**
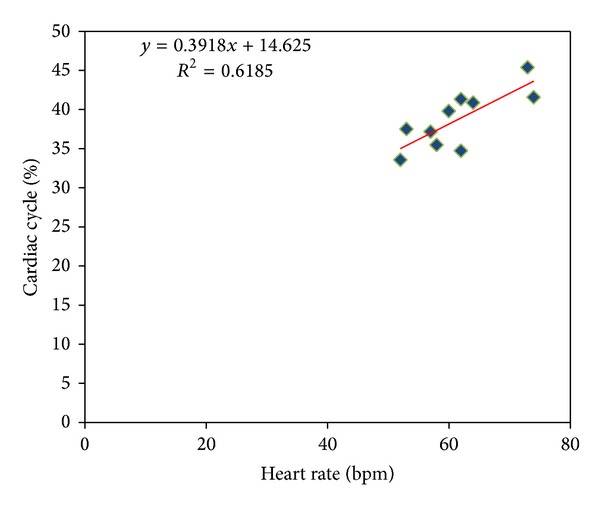
The regression of cardiac cycle percentage and heart rate for 10 healthy subjects as well as best linear fit trend lines.

The R-AC delay in each cardiac cycle was measured to compute percentage of the cardiac cycle as calculated by [4]. The cardiac cycle percentage was found by dividing each R-AC into its corresponding cardiac cycle length (R-R interval) [4]. A linear correlation between the cardiac cycle percentage and heart rate was observed. Pearson correlation coefficient between the cardiac cycle percentage and heart rate was found high (*r* = 0.79).  Figure 10 shows the scatter plot of the linear relationship between the mean cardiac cycle percentage and the heart rate during normal breathing with all test subjects.

**Figure 11:**
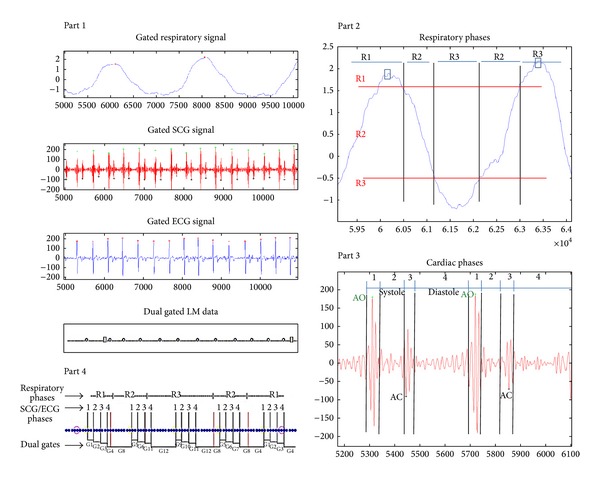
Prospective dual gating diagrams acquired from accelerometer-based method.

Figure 11 illustrates a prospective dual gating method using SCG signal. This diagram shows a corresponding diagram related to list mode data method as developed by [20] for dual gating method. Figure 11 also shows that the gated respiratory motion data extracted from the accelerometer has potential to be used instead of RPM systems in nuclear medicine imaging. The solid red signal refers to the SCG signal (Figure 11 (Part 1)). The AO peaks are recognizable by green markers (Figure 11 (Part 2)). The solid blue signal marked by red markers refers to ECG signal (Figure 11 (Part 3)).

As mentioned earlier, in comparison with ECG, the cardiac events were detected more accurately by SCG. Therefore, in order to obtain better phases for gating the image acquisition, it can be proposed that SCG might provide accurate triggering pulses.

Consequently, the accelerometer-based technique represents respiratory cycles according to their amplitude and duration as well as validating the cardiac cycle length associated with the ECG.

The principles of the list mode (LM) dual gating are originated according to [5, 6, 21] as follows.

**Part 1 .**

Gated respiratory motion data from SCG file, gated SCG and ECG signals, and LM data with triggers: open square refers to respiratory trigger, open circle turns to SCG trigger, and dot determines the LM data event.

**Part 2 .**

Respiratory phases determined by amplitude signal: R1 refers to inspiration, R2 turns to middle, and R3 turns to expiration. Horizontal red lines indicate splitting of respiration cycle into three phases (R1–R3). Vertical black lines indicate onsets of respiratory phases (R1–R3).

**Part 3 .**

SCG phases: phases 1 to 3 are constant systole intervals and phase 4 is time-varying diastole interval. Vertical black lines (1–4) indicate onsets of ECG phases.

**Part 4 .**

Dual gated LM data: vertical red and black lines indicate respiratory (R1–R3) and SCG (1–4) labels on LM data. Dual gates (G1–G12) are determined by respiratory and SCG phase labels. Dual gated LM data are divided into 12 different LM files.

As the results were indicated in this study, determined cardiac intervals across both accelerometer-based and ECG-based methods were detected accurately using the developed algorithm. However, the proposed method for cardiac gating requires more studies. To use the algorithm for cardiac gating, some issues must be considered as follows.

Firstly, an advanced algorithm is essential to detect the cardiac cycles as well as amplitude and duration of respiratory cycles across the SCG and ADR signals.

The algorithm should work reliably for everybody, because, in real nuclear medicine imaging, only the people who are suffering from the cardiovascular disease will undergo the X-ray radiations. For instance, cardiac arrhythmia might make some difficulties for ECG-gating technique, whereas in the accelerometer-based technique irregular cardiac rhythms were not considered. Thus, to represent an accelerometer-based solution for detecting cardiac intervals correctly, the gating algorithm is required to assess the applicability of the detected intervals for the cardiac gating based on acceptable sinus arrhythmia and heart rate variability range.

Secondly, due to the long signal processing time caused by large amounts of data in gating nuclear medicine imaging, the current accelerometer-based method could not be utilized in real-time cardiac, respiratory, and dual gating. However, it is possible to improve the measurement and processing units in the future.

In this study, the segregation of the cardiac signals and datasets was accomplished using downsampling, median filter, and moving-average filter in the time domain. Moreover, the excellent correlation and high agreement between accelerometer-based measurement and reference measurements establish that reliable respiratory and cardiac gating signals could be extracted using a single triaxis accelerometer. As can be seen from Figure 7, a reliable respiratory gating signal with excellent correlation was acquired regardless of the respiratory depth and rates with all test subjects. Besides, a good cardiac gating signal was obtained from all test subjects with fairly normal sinus rhythm across the normal breathing part.

The statistical analysis and acquired results of this project prove that seismocardiography and ADR signals validate the hypothesis of this study which was implementation of the accelerometer-based technique in nuclear medicine imaging.

**Figure 12:**
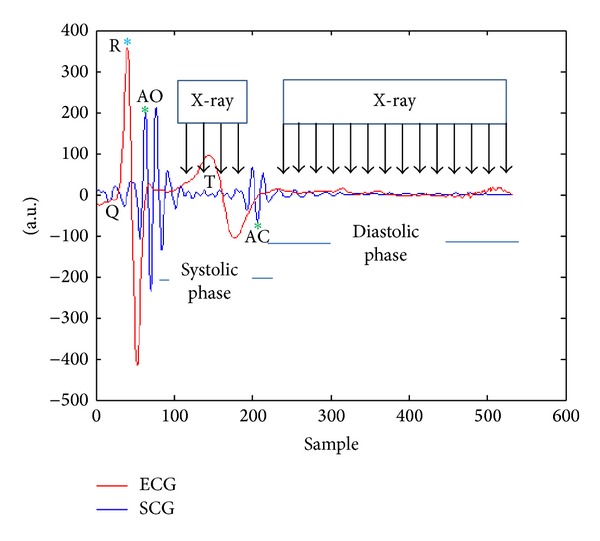
Tighter padding intervals for prospective ECG gating.

Figure 12 implies that, by performing accelerometer-based method before prospective ECG gating for CT coronary angiography (CTCA), tighter padding intervals could be used which results in better management for radiation of the X-ray beam through the patient body [4].

Reference [22] reported that the prospective cardiac gating decreases patient radiation doses by managing the X-ray beam on and off time during motion-free phases of cardiac cycle.

Consequently, the gated image acquisition for cardiac studies and oncological studies near the diaphragm will decrease motion artifacts and radiation doses as well as increasing image quality in nuclear medicine imaging [23]. Furthermore, the accelerometer-based gating method might be achievable in MRI imaging as well as in radiotherapy in the future. However, for MRI and radiotherapy purposes further development of the accelerometer-based method is critical as well. For instance, in MRI studies, the data acquisition systems, accelerometer sensor, electrodes, and cables have to be MR compatible.

There are some limitations in this study such as the following. Only healthy people were examined while real patients suffering cardiovascular disease are excluded. Moreover, measurements are not performed during cardiac imaging session such as PET. The SCG represents left ventricular activity (e.g., AO and AC), because normally the muscle mass of the left ventricle is about three times that of the right ventricle, so if there was any problem in the right ventricle or other heart valves, probably SCG has some limitations. Physiological measurements were not performed during this study such as left ventricular ejection fraction with the use of SCG technology, and no real PET/CT imaging was prepared in this study.

## 5. Conclusion

The accelerometer-based system is a potential solution for dual gating in nuclear medicine imaging. It would ease the work of technologists as both gating signals can be acquired with a single device. Besides, it was investigated in the prestudy of this thesis that the PET imaging equipment does not interfere with the accelerometer signal during image acquisition. Moreover, the equipment is familiar to the technologist, since essentially similar devices are used in ECG gating. The method is also comfortable for the patients as only standard sensor and electrodes are needed for its implication.

Overall, the proposed system might offer motionless images with the lower radiation dose. Having a high resolution cardiac image definitely helps a heart specialist in making a correct diagnosis as well as planning a sufficient therapy. Future studies are needed to investigate the real patient who is suffering from irregular heart arrhythmia or coronary artery diseases as well as investigation of accelerometer-based method versus real-time position management (RPM) system, spirometer, and bioimpedance based method. Besides, the sensitivity of this method should be assessed by recruiting more BMI (obese) subjects with or without high heart rates. Further investigation is needed to identify the radiation opaquely of the accelerometer in order to apply this method for real nuclear medicine imaging procedures.

## Figures and Tables

**Table 1:** Mean and standard deviation (σ) of the breathing rate from all subjects for the requested patterns.

	Normal	Slow	Fast
Mean (bpm)	11.1	6.7	23.3
σ	1.72884	1.828782	8.870049
